
JOURNAL INFORMATION
==============================
NLM Title Abbreviation: J Vet Intern Med
Journal ID: J Vet Intern Med
NLM Journal ID: 8708660
Journal ID: JVIM

Journal of Veterinary Internal Medicine

ISSN: 0891-6640
EISSN: 1939-1676
Publisher: Oxford University Press

ARTICLE INFORMATION
==============================
PMCID: PMC11474532
DOI: 10.1111/jvim.16761
Article ID: JVIM16761
Article version: 1
Subjects: Issue Information, Issue Information

Issue Information

Electronic publication date: 2024 May 16
Publication date: 2024 May-Jun
Volume: 38
Issue: 3
First page: 1273
Last page: 1281
Copyright: © 2024 The Authors. Journal of Veterinary Internal Medicine published by Wiley Periodicals LLC on behalf of American College of Veterinary Internal Medicine.
License: This is an open access article under the terms of the http://creativecommons.org/licenses/by/4.0/ License, which permits use, distribution and reproduction in any medium, provided the original work is properly cited.
License URL: https://creativecommons.org/licenses/by/4.0/

Figure count: 0
Table count: 0
Page count: 1

source-schema-version-number: 2.0
cover-date: May/June 2024
details-of-publishers-convertor: Converter:WILEY_ML3GV2_TO_JATSPMC version:6.4.9 mode:remove_FC converted:15.10.2024

==============================

Volume 38, Number 3 May/June 2024

### JOURNAL OF VETERINARY INTERNAL MEDICINE

Open Access

ACVIM
American College of Veterinary Internal Medicine

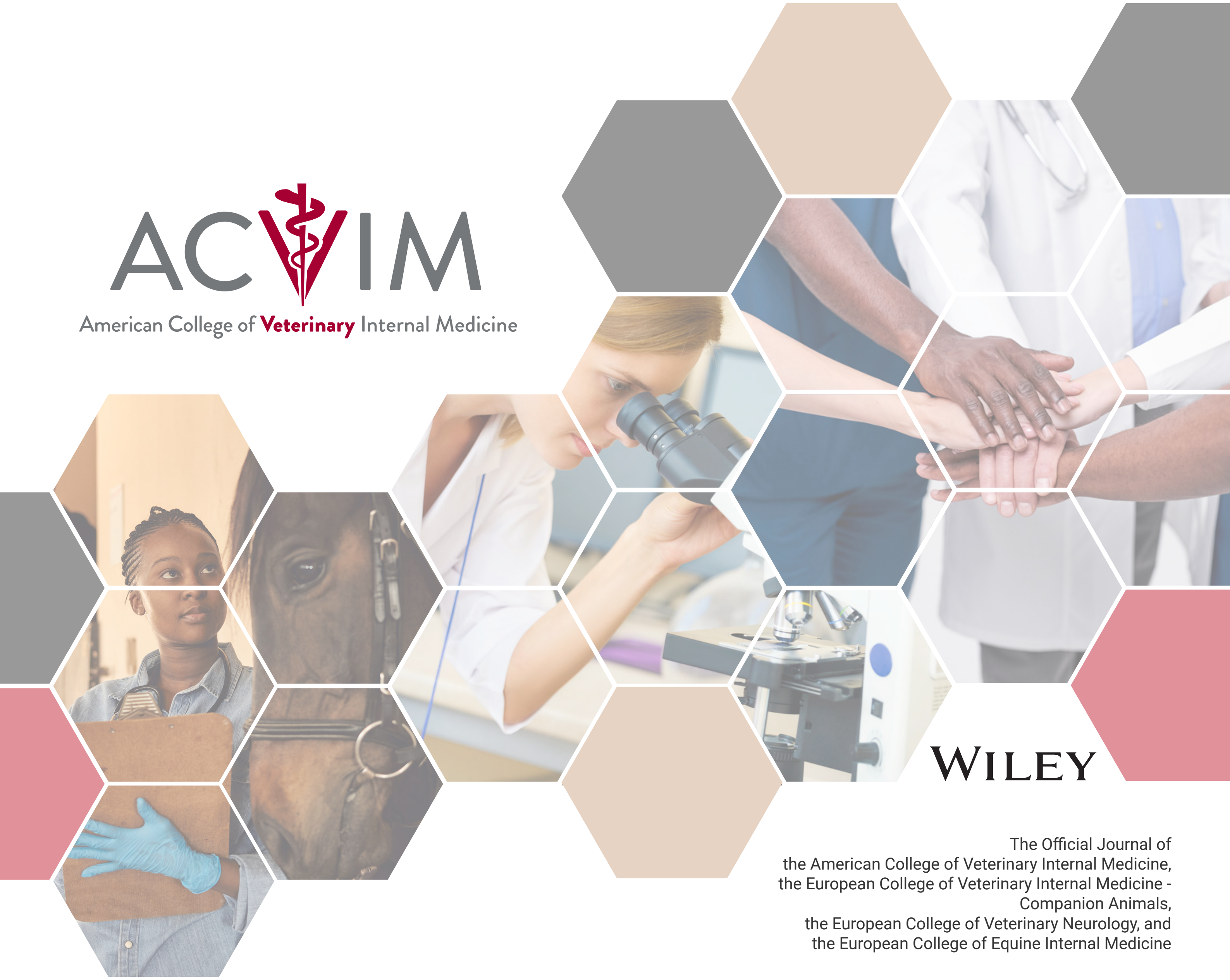

WILEY

The Official Journal of
the American College of Veterinary Internal Medicine,
the European College of Veterinary Internal Medicine - Companion Animals,
the European College of Veterinary Neurology, and
the European College of Equine Internal Medicine

### Journal of Veterinary Internal Medicine

Open Access

ACVIM
American College of Veterinary Internal Medicine

The Official Journal of the American College of Veterinary Internal Medicine,
the European College of Veterinary Internal Medicine - Companion Animals, the European College of Veterinary Neurology,
and the European College of Equine Internal Medicine

**MISSION STATEMENT**

The mission of the *Journal of Veterinary Internal Medicine* is to advance veterinary medical knowledge and improve the lives of animals by publication of authoritative scientific articles of animal diseases.

**Volume 38, Number 3** **May/June 2024**

LETTERS TO THE EDITOR

1282 **Letter regarding "Fuzapladib in a randomized controlled multicenter masked study in dogs with presumptive acute onset pancreatitis"**
*Karin Allenspach, Andreas Handel, Stanley Marks, Peter Kook, Kenny Simpson, Joseph Bartges, Kenjiro Fukushima, Aarti Kathrani, Silke Salavati, Julien Dandrieux, Caroline Mansfield, Alison Manchester, Craig Webb, Valerie Freiche, W. Zane Billings, and Jonathan P. Mochel*

1285 **Response to letter regarding "Fuzapladib in a randomized controlled multicenter masked study in dogs with presumptive acute onset pancreatitis"**
*Jörg M. Steiner, Chantal Lainesse, Yuya Noshiro, Yumiko Domen, Heather Sedlacek, Stephen E. Bienhoff, Kelly P. Doucette, David L. Bledsoe, and Hiroshi Shikama*

1287 **Letter regarding "Evaluation of the clinical outcome of hypercalcemia of malignancy and concurrent azotemia in dogs with lymphoma"**
*C. Guillermo Couto and Dennis J. Chew*

1289 **Response to letter regarding "Evaluation of the clinical outcome of hypercalcemia of malignancy and concurrent azotemia in dogs with lymphoma"**
*Alyssa A. Strumpf, Laura Selmic, and Brian Husbands*

CONSENSUS STATEMENT

1290 **Updated ACVIM consensus statement on equine herpesvirus-1**
*David P. Lunn, Brandy A Burgess, David C. Dorman, Lutz S. Goehring, Peggy Gross, Klaus Osterrieder, Nicola Pusterla, and Gisela Soboll Hussey*

SMALL ANIMAL

Standard Articles

*Cardiology*

1300 **Echocardiographic and electrocardiographic findings in Irish Wolfhounds eating high-pulse or low-pulse diets**
*Lily M. Coppinger, Lisa M. Freeman, William D. Tyrrell Jr, Steven L. Rosenthal, Mariellen E. Dentino, Frances L. Abrams, and John E. Rush*

1305 **Use of machine learning and Poincaré density grid in the diagnosis of sinus node dysfunction caused by sinoatrial conduction block in dogs**
*Wyatt Hutson Flanders, N. Sydney Moïse, and Niels F. Otani*

1325 **Utility of focused cardiac ultrasonography training in veterinary students to differentiate stages of subclinical myxomatous mitral valve disease in dogs**
*Terry Huh, Erin J. Achilles, Laura K. Massey, Kerry A. Loughran, Éva Larouche-Lebel, Victoria Convey, Victoria F. McKaba, Alexandra Crooks, Marc S. Kraus, Anna R. Gelzer, and Mark A. Oyama*

*Endocrinology*

1334 **Evaluation of muscle mass and intramuscular fatty infiltration in dogs with hypercortisolism and their association with prognosis**
*Kei Yoshida, Yui Kobatake, Satoshi Takashima, and Naohito Nishii*

1345 **Total thyroxine, triiodothyronine, and thyrotropin concentrations during acute nonthyroidal illness and recovery in dogs**
*Andrea Corsini, Francesca Del Baldo, Francesco Lunetta, Serena Ribichini, Massimo Giunti, Francesca Fidanzio, and Federico Fracassi*

1353 **Effect of sodium-glucose cotransporter 2 inhibitor canagliflozin on interstitial glucose concentration in insulin-treated diabetic dogs**
*Jessica R. Box, Mark A. Oyama, Ariel S. Mosenco, and Rebecka S. Hess*

1359 **Blood pressure in hyperthyroid cats before and after radioiodine treatment**
*Lisa Stammeleer, Pilar Xifra, Sara I. Serrano, Mark Rishniw, Sylvie Daminet, and Mark E. Peterson*

1370 **Effects of prednisolone on 1,2-O-dilauryl-rac-glycero glutaric acid-(60-methylresorufin) ester-lipase activity and pancreatic lipase immunoreactivity in healthy cats**
*Militsa Pacheva, Daniel Brugger, Barbara Riond, Matthias Dennler, and Peter Hendrik Kook*

1377 **Assessment of hemostasis in hyperthyroid and euthyroid cats using two viscoelastic assays and platelet aggregometry**
*Daniel Moreno, Kevin Cosford, Elisabeth Snead, and Anthony Carr*

1384 **Development and validation of a questionnaire to assess health-related quality-of-life in cats with hyperthyroidism**
*Fabienne Blunschi, Imogen Schofield, Sofie Muthmann, Natali B. Bauer, and Katarina Hazuchova*

*Genetics*

1408 **A novel *ITGA2B* double cytosine frameshift variant (c.1986_1987insCC) leads to Glanzmann's thrombasthenia in a cat**
*Victor N. Rivas, Avalene W. K. Tan, Meg Shaverdian, Nghi P. Nguyen, Jalena R. Wouters, Joshua A. Stern, and Ronald H. L. Li*

1418 **A de novo nonsense variant in the *DMD* gene associated with X-linked dystrophin-deficient muscular dystrophy in a cat**
*Nozomu Yokoyama, Yuki Matsumoto, Takahisa Yamaguchi, Kazuki Okada, Ryohei Kinoshita, Genya Shimbo, Hisashi Ukawa, Ryuga Ishii, Kensuke Nakamura, Jumpei Yamazaki, and Mitsuyoshi Takiguchi*

*Gastroenterology*

1425 **Safety profile and effects on the peripheral immune response of fecal microbiota transplantation in clinically healthy dogs**
*Mary Ann Lee, Maria Questa, Patrawin Wanakumjorn, Amir Kol, Bridget McLaughlin, Bart C. Weimer, Agostino Buono, Jan S. Suchodolski, and Sina Marsilio*

1437 **Peripheral and intestinal T lymphocyte subsets in dogs with chronic inflammatory enteropathy**
*Beatriz Agulla, Alejandra Villaescusa, Ángel Sainz, David Díaz-Regañón, Fernando Rodríguez-Franco, Lydia Calleja-Bueno, Patricia Olmeda, and Mercedes García-Sancho*

1449 **Incidence and characterization of aerophagia in dogs using videofluoroscopic swallow studies**
*Megan Grobman, Carol Reinero, Tekla Lee-Fowler, and Teresa E. Lever*

1458 **Comparison of hematologic variables between dogs with congenital intrahepatic and extrahepatic portosystemic shunts**
*Yanick Couture, Deborah Keys, and Stacie Summers*

1465 **The effect of feeding on serum concentrations of cobalamin, folate, trypsin-like immunoreactivity, and pancreatic lipase immunoreactivity in dogs with signs of chronic gastrointestinal disease**
*Ashley Melco, Jessica C. Pritchard, Scott J. Hetzel, Alexander Saver, Joao Pedro Cavasin, and Jörg M. Steiner*

1475 **Evaluating acidic gastroesophageal reflux with wireless pH monitoring in French bulldogs with sliding hiatal herniation**
*Tarini V. Ullal, Stanley L. Marks, Nuen Tsang Yang, and Sandra L. Taylor*

*Hematology*

1483 **Thromboelastometry for assessment of hemostasis and disease severity in 42 dogs with naturally-occurring heatstroke**
*Michal Yanai, Sigal Klainbart, Gal Dafna, Gilad Segev, Itamar Aroch, and Efrat Kelmer*

1498 **Effect of leukoreduction on the omics phenotypes of canine packed red blood cells during refrigerated storage**
*Arianna Miglio, Francesca Rocconi, Valentina Cremoni, Angelo D'Alessandro, Julie A. Reisz, Mark Maslanka, Ian S. Lacroix, Daniela Di Francesco, Maria T. Antognoni, and Morena Di Tommaso*

1512 **Utility of immature platelet fraction in the Sysmex XN-1000V for the differential diagnosis of central and peripheral thrombocytopenia in dogs and cats**
*Alejandro Perez-Ecija, Carmen Martinez, Julio Fernandez-Castañer, and Francisco J. Mendoza*

*Infectious Disease*

1520 **Evaluation of antimicrobial purchasing by companion animal veterinary facilities in Canada, the United Kingdom, and the United States of America (2019-2021)**
*J. Scott Weese, Margo Mosher, Rochelle Low, Ellie West, Ben O'Kelley, Jo Ann Morrison, Anne Kimmerlein, Silene St. Bernard, Kathrine Blackie, Ulrika Gronlund, and Ian Battersby*

1535 **Prognostic potential of copper, zinc, copper/zinc ratio, cobalamin, and serum amyloid A in cats with panleukopenia**
*Kerim Emre Yanar, Sümeyye Baysal, Nergis Ulaş, Mustafa Sinan Aktaş, Mehmet Özkan Timurkan, and Hakan Aydın*

*Nephrology/Urology*

1542 **Usefulness of serum amyloid A for the diagnosis of pyelonephritis in cats: A prospective evaluation**
*Maxime Kurtz, Pascaline Bénédicte Marie Pey, Jérémy Mortier, Mathieu Manassero, Fiona Da Riz, Morgane Canonne-Guibert, Christelle Maurey, and Ghita Benchekroun*

1553 **Detection of nephrocalcinosis using ultrasonography, micro-computed tomography, and histopathology in cats**
*Pak-Kan Tang, Rebecca F. Geddes, Yu-Mei Chang, Rosanne E. Jepson, Dirk Hendrik Nicolaas van den Broek, Nicola Lötter, and Jonathan Elliott*

1563 **Risk factors and implications associated with ultrasound-diagnosed nephrocalcinosis in cats with chronic kidney disease**
*Pak-Kan Tang, Rebecca F. Geddes, Yu-Mei Chang, Rosanne E. Jepson, Dirk Hendrik Nicolaas van den Broek, Nicola Lötter, and Jonathan Elliott*

*Neurology*

1577 **Evaluation of neurofilament light chain as a biomarker in dogs with structural and idiopathic epilepsy**
*Kayla M. Fowler, Richard L. Shinn, John H. Rossmeisl, and Rell L. Parker*

1583 **Prognosis in meningoencephalitis of unknown origin in dogs: Risk factors associated with survival, clinical relapse, and long-term disability**
*Rita Gonçalves, Steven De Decker, Gemma Walmsley, and Thomas W. Maddox*

1591 **Clinical features and outcome of 10 dogs with suspected idiopathic vestibular epilepsy**
*Tania Al Kafaji, Fabio Tocco, Samuel Okonji, and Antonella Gallucci*

1598 **Prevalence, clinical presentation, and etiology of myelopathies in 224 juvenile dogs**
*Ed J. Pilkington, Steven De Decker, Eleftheria Skovola, Ana Cloquell Miro, Rodrigo Gutierrez Quintana, Kiterie M. E. Faller, Albert Aguilera Padros, and Rita Goncalves*

1608 **Influence of body weight, age, and sex on cerebrospinal fluid peak flow velocity in dogs without neurological disorders**
*Johannes Rich, Sarah Hubler, Beatriz Vidondo, Mathieu Raillard, and Daniela Schweizer*

1618 **Corticosteroid monotherapy versus combined cytarabine continuous rate infusion and corticosteroid therapy in dogs with meningoencephalitis of unknown origin: A blinded, randomized, controlled trial**
*Bethan S. Jones, Francois Xavier Liebel, Angela Fadda, Sophie Martin, Richard Lawn, Kali Lazzerini, and Thomas Harcourt-Brown*

1626 **Traumatic and iatrogenic sciatic nerve injury in 38 dogs and 10 cats: Clinical and electrodiagnostic findings**
*Diletta Dell'Apa, Luigi Auletta, Samuel Okonji, Alberto Cauduro, Maurizio Dondi, Mauro Opreni, Gualtiero Gandini, and Ezio Bianchi*

1639 **Client's understanding of instructions for small animals in a veterinary neurological referral center**
*Thomas Flegel, Katharina Dobersek, Sabrina Bayer, Lisa F. Becker, Shenja Loderstedt, Irene C. Böttcher, Josephine Dietzel, Carina Tästensen, Theresa Kalliwoda, Marie A. Harkenthal, Andreas Kühnapfel, Vivian Weiß, and Sarah Gutmann*

*Oncology*

1651 **Comparison of chemotherapy outcomes between normal and high serum cortisol concentration in dogs with lymphoma**
*Hiroki Yamazaki, Kaito Bunbai, Tatsuya Deguchi, Masahiro Tamura, and Hiroshi Ohota*

1659 **Environmental radon, fracking wells, and lymphoma in dogs**
*Ashleigh N. Tindle, Samantha L. Braman, Brenna M. Swafford, and Lauren A. Trepanier*

1666 **Safety and biologic activity of a canine anti-CD20 monoclonal antibody in dogs with diffuse large B-cell lymphoma**
*Gretchen P. McLinden, Anne C. Avery, Heather L. Gardner, Kelley Hughes, Angie M. Rodday, Kexuan Liang, and Cheryl A. London*

1675 **Weishaar's classification system for nodal metastasis in sentinel lymph nodes: Clinical outcome in 94 dogs with mast cell tumor**
*Damiano Stefanello, Elisa M. Gariboldi, Patrizia Boracchi, Roberta Ferrari, Alessandra Ubiali, Donatella De Zani, Davide D. Zani, Valeria Grieco, Chiara Giudice, Camilla Recordati, Mario Caniatti, Luigi Auletta, and Lavinia E. Chiti*

1686 **Vincristine-induced adverse events related to body weight in dogs treated for lymphoma**
*Keira E. Sztukowski, Zachary Yaufman, Matthew R. Cook, Turi K. Aarnes, and Brian D. Husbands*

1693 **Descriptive analysis and prognostic factors in cats with myeloma-related disorders: A multicenter retrospective study of 50 cases**
*Lorris Lecot, Isabelle Desmas-Bazelle, Sarah Benjamin, Pauline De Fornel, Frédérique Ponce, Matthew Kornya, Loïc Desquilbet, Claire Beaudu-Lange, Catherine Ibisch, David Sayag, Ghita Benchekroun, and Jérémy Béguin*

*Pharmacology*

1706 **Effect of institutional antimicrobial stewardship guidelines on prescription of critically important antimicrobials for dogs and cats**
*Sarah N. Robbins, Robert Goggs, Sydney Kraus-Malett, and Laura Goodman*

*Respiratory*

1718 **Assessing breathing effort by barometric whole-body plethysmography and its relationship with prognosis in client-owned cats with respiratory distress**
*Wei-Tao Chang, Chung-Hui Lin, Chin-Hao Chang, Pei-Ying Lo, Hui-Wen Chen, and Huey-Dong Wu*

Case Reports

*Infectious Disease*

1725 **Positive clinical outcome using a modified dosing regimen of benznidazole in dogs at high risk for infection or acutely infected with *Trypanosoma cruzi***
*Sukjung Lim, Stephanie Collins, Sarah A. Hamer, Rick L. Tarleton, and Ashley B. Saunders*

*Neurology*

1730 **Congenital spongiform leukodystrophy in 2 female littermate German shepherd puppies**
*Ricardo De Miguel, Devon Wallis Hague, Jennifer L. Johnson, Amber M. Zilinger, Anna Kukekova, and Stephane Lezmi*

1737 **Clinical and magnetic resonance imaging findings in a French bulldog puppy with genetically confirmed congenital hypothyroidism**
*Katia Sánchez González, Harry Warwick, Marius Conradie, and Neringa Alisauskaite*

*Oncology*

1744 **Malignant transformation and subsequent leptomeningeal carcinomatosis of a gastric polyp in a dog**
*Dillon S. Didehvar, Matthew R. Lanza, Matthew J. Atherton, and Jennifer A. Lenz*

**EQUINE**

Standard Articles

*Cardiology*

1751 **Twenty-four hour continuous transvenous temporary right ventricular pacing in healthy horses**
*Amanda Avison, Anna R. Gelzer, Virginia B. Reef, Kathryn B. Wulster Bills, Cris Navas de Solis, Marc S. Kraus, JoAnn Slack, Darko Stefanovski, Lindsay J. Deacon, and Claire Underwood*

*Infectious Disease*

1765 **Viremia and nasal shedding for the diagnosis of equine herpesvirus-1 infection in domesticated horses**
*Nicola Pusterla, David C. Dorman, Brandy A. Burgess, Lutz Goehring, Margaret Gross, Klaus Osterrieder, Gisela Soboll Hussey, and David P. Lunn*

*Neurology*

1792 **Equine neuroaxonal dystrophy/degenerative myeloencephalopathy in Gypsy Vanner horses**
*Alexis Powers, Simon F. Peek, Steve Reed, Callum G. Donnelly, Stacey Tinkler, David Gasper, Kevin D. Woolard, and Carrie J. Finno*

1799 **Long-term performance of show-jumping horses and relationship with severity of ataxia and complications associated with myeloencephalopathy caused by equine herpes virus-1**
*María de la Cuesta-Torrado, Ana Velloso Alvarez, Patricia Neira-Egea, and Juan Cuervo-Arango*

1808 **Clinicopathological and pedigree investigation of a novel spinocerebellar neurological disease in juvenile Quarter Horses in North America**
*Andrew T. Willis, Anna R. Dahlgren, Kevin D. Woolard, Sharmila Ghosh, Callum G. Donnelly, Andres de la Concha-Bermejillo, Ana Pacheco, Katherine D. Watson, Emily Berryhill, Monica Aleman, Fiona Wensley, Sarah Humphreys, Ashley E. Whitehead, Dayna Goldsmith, Berkley Chesen, John Ragsdale, James E. Tompkins, Ron Nash, Amanda H. Plunkett, Heath J. Qualls, Katarina Rodriguez, Damaris Hochanadel, Andrew D. Miller, and Carrie J. Finno*

*Oncology*

1815 **A randomized placebo-controlled double-blinded study comparing oral and subcutaneous administration of mistletoe extract for the treatment of equine sarcoid disease**
*Anke Beermann, Ophélie Clottu, Marcus Reif, Ulrike Biegel, Lucia Unger, and Christoph Koch*

*Pharmacology*

1825 **Pharmacokinetics of amikacin after intravenous, intra-articular, and combined intravenous and intra-articular administration in healthy neonatal foals**
*Jillian L. Paegelow, Mike J. Schoonover, Jenna M. Young, Lara K. Maxwell, Jared D. Taylor, Lyndi L. Gilliam, and Todd C. Holbrook*

*Respiratory*

1835 **Comparative study of the bronchodilator efficacy and adverse effects of salbutamol and hyoscine butylbromide in horses with severe asthma**
*Berta Mozo Vives, Sophie Mainguy-Seers, and Jean-Pierre Lavoie*

1842 **Clinical findings and outcome predictors for multinodular pulmonary fibrosis in horses: 46 cases (2009-2019)**
*Amanda Craven, Amy Todd-Donato, Tracy Stokol, Rachel Liepman, Ilana Glasberg, Pamela Wilkins, Daniela Luethy, David Wong, Angelika Schoster, Astrid J. van den Brom - Spierenburg, and Joy E. Tomlinson*

Systematic Review

*Infectious Disease*

1858 **Vaccination for the prevention of equine herpesvirus-1 disease in domesticated horses: A systematic review and meta-analysis**
*Klaus Osterrieder, David C. Dorman, Brandy A. Burgess, Lutz S. Goehring, Peggy Gross, Claire Neinast, Nicola Pusterla, Gisela Soboll Hussey, and David P. Lunn*

1872 **Relationship between equine herpesvirus-1 viremia and abortion or equine herpesvirus myeloencephalopathy in domesticated horses: A systematic review**
*Gisela Soboll-Hussey, David C. Dorman, Brandy A. Burgess, Lutz Goehring, Peggy Gross, Claire Neinast, Klaus Osterrieder, Nicola Pusterla, and David P. Lunn*

1892 **Pharmacologic interventions for the treatment of equine herpesvirus-1 in domesticated horses: A systematic review**
*Lutz Goehring, David C. Dorman, Klaus Osterrieder, Brandy A. Burgess, Kelsie Dougherty, Peggy Gross, Claire Neinast, Nicola Pusterla, Gisela Soboll-Hussey, and David P. Lunn*

**FOOD ANIMAL**

Standard Articles

*Hematology*

1906 **Bayesian evaluation of sensitivity and specificity of blood culture media and hypoglycemia in sepsis-suspected calves**
*Mathilde Laetitia Pas, Filip Boyen, Donatienne Castelain, Laurens Chantillon, Dominique Paepe, Frederik Pille, Bart Pardon, and Jade Bokma*

*Neurology*

1917 **Prognostic indicators of outcome in nonambulatory beef cattle presented to 2 referral hospitals: A retrospective study of 63 cases**
*Diana Perez-Solano, Emily J. Reppert, Jenna E. Stockler, Pankaj Gaonkar, Laura Huber, Thomas Passler, Jenna E. Bayne, and Manuel F. Chamorro*

1925 **Clinical findings and outcome in goats with discospondylitis and vertebral osteomyelitis**
*Alyssa Sullivan, Elodie Huguet, Ashley R. VanderBroek, Shannon Darby, and Daniela Luethy*

*Respiratory*

1932 **Blood gases, acid-base, and metabolic alterations in calves with bronchopneumonia diagnosed via clinical signs and thoracic ultrasonography: A cross-sectional study**
*Antonio Boccardo, Vincenzo Ferrulli, Giulia Sala, Donatella Scavone, Saverio Paltrinieri, Laura Filippone Pavesi, and Davide Pravettoni*

1941 **Randomized comparison between a forced air system and warm water bath for resuscitation of neonatal hypothermic calves with or without oral administration of caffeine**
*Adam T. Copeland, Amanda J. Kreuder, Grant Dewell, Renee Dewell, Caitlin Wiley, Lingnan Yuan, Jonathan P. Mochel, and Joe S. Smith*

### Journal of Veterinary Internal Medicine

Open Access

ACVIM American College of Veterinary Internal Medicine

The Official Journal of the American College of Veterinary Internal Medicine,
the European College of Veterinary Internal Medicine - Companion Animals, the European College of Veterinary Neurology, and the European College of Equine Internal Medicine

### EDITORIAL BOARD

Kenneth W. Hinchcliff, BVSc, PhD, DACVIM
Editor-in-Chief
The University of Melbourne
Stephen P. DiBartola, DVM, DACVIM
Editor-in-Chief
The Ohio State University
Joshua Stern, DVM, PhD, DACVIM
Associate Editor - Cardiology
North Carolina State University
Jose Novo Matos, DVM, PhD, DECVIM-CA, AFHEA, MRCVS
Associate Editor - Small Animal Internal Medicine, Cardiology
University of Cambridge
Mark Stevenson, MVSc, PhD, MANZCVSc
Consulting Editor for Experimental Design & Statistics
The University of Mebourne
George Moore, DVM, MS, PhD, DACVIM
Consulting Editor for Experimental Design & Statistics
Purdue University
Kristopher J. Hughes, BVSc (Hons 1), FANZCVS, DECEIM, Associate ACVIM
ECEIM Associate Editor - Large Animal Internal Medicine (Equine)
Charles Sturt University
Linda Kidd, DVM, PhD, DACVIM
Associate Editor - Small Animal Internal Medicine (Infectious Disease)
Linda Kidd Veterinary Internal Medicine Consulting
M. Julia B. Felippe, MV, MSc, PhD, DACVIM
Associate Editor - Large Animal Internal Medicine (Equine)
Cornell University
Shelly L. Vaden, DVM, PhD, DACVIM
Associate Editor - Small Animal Internal Medicine (Nephrology/Urology)
North Carolina State University
Michael Willard, DVM, DACVIM
Associate Editor - Small Animal Internal Medicine
Texas A&M University
Andrea Tipold, DVM, DECVN
ECVN Associate Editor - Neurology
School of Veterinary Medicine Hannover
Robert Rebhun, DVM, PhD, DACVIM
Associate Editor - Hematology/Oncology
University of California, Davis
Eric Zini, PD, PhD, DECVIM-CA
ECVIM-CA Associate Editor - Small Animal Internal Medicine (Endocrinology)
University of Zurich & Istituto Veterinario di Novara & University of Padua
Munashe Chigerwe, BVSc, MPH, PhD, DACVIM
Associate Editor - Large Animal Internal Medicine (Food & Fiber)
University of California, Davis
Lauren Trepanier, DVM, PhD, DACVCP, DACVIM
Associate Editor – Small Animal Internal Medicine (Pharmacology, Pharmacokinetics, Toxicology, and Therapeutic Trials)
University of Wisconsin
Valerie Parker, DVM, DACVIM
Associate Editor - Small Animal Internal Medicine, Nutrition
The Ohio State University
Steven Friedenberg, DVM, PhD, DACVECC
Associate Editor - Small Animal Internal Medicine (Genetics, Genomics)
University of Minnesota
Anthony Mutsaers, DVM, PhD, DACVIM
Associate Editor - Oncology
University of Guelph
John Farrelly, DVM, DACVIM, DACVR
Associate Editor - Oncology
Blue Pearl Veterinary Partners

**ACVIM Board of Regents Officers**

Joerg Steiner, DMV, PhD, DACVIM, DECVIM-CA
Board Chair
Sarah Gillings, DVM, MS, DACVIM
President
Bill Tyrrell, DVM, DACVIM
Treasurer

**Ex-Officio Member of the ACVIM Board of Regents**

Jane Sykes, BVSc (Hons), PhD, MBA, DACVIM
Immediate Past Board Chair

**The Editorial and ACVIM Offices are located at:**

116 Inveress Drive East, Suite 375, Englewood, CO 80112

**Telephone:** 303-231-9933 or 800-245-9081

**Editorial Email:** JVIM@ACVIM.org.
**ACVIM Email:** ACVIM@ACVIM.org.

The Journal is available online at www.wileyonlinelibrary.com/journal/jvim.

**ACVIM Board of Regents Members**

Brent Credille, DVM, PhD, DACVIM
Member At-Large
Tracy Hill, DVM, DACVIM, PhD, DECVIM-CA
Member At-Large
Roger A. Hostutler, DVM, MS, DACVIM
President, Small Animal Internal Medicine
Chris Sanchez, DVM, PhD, DACVIM
President, Large Animal Internal Medicine
Steven L. Rosenthal, DVM, DACVIM
President, Cardiology
Joan R. Coates, DVM, MS, DACVIM
President, Neurology
Chad Johannes, DVM, DACVIM
President, Oncology
Shannon Carter, EdD, CAE
Chief Executive Officer

### Journal of Veterinary Internal Medicine

Open Access

ACVIM American College of Veterinary Internal Medicine

The Official Journal of the American College of Veterinary Internal Medicine,
the European College of Veterinary Internal Medicine - Companion Animals, the European College of Veterinary Neurology, and the European College of Equine Internal Medicine

Wiley Open Access articles posted to repositories or websites are without warranty from Wiley of any kind, either express or implied, including, but not limited to, warranties of merchantability, fitness for a particular purpose, or non-infringement. To the fullest extent permitted by law Wiley disclaims all liability for any loss or damage arising out of, or in connection with, the use of or inability to use the content.

**Open Access and Copyright**

All articles published by *Journal of Veterinary Internal Medicine* are fully open access: immediately freely available to read, download and share. All articles are published under the terms of a Creative Commons license. Copyright on any research article published in the *Journal of Veterinary Internal Medicine* is retained by the author(s).

Further information about open access licenses and copyright can be found at: https://authorservices.wiley.com/author-resources/Journal-Authors/licensing/licensing-info-faqs.html#4. Information on the licenses used by *Journal of Veterinary Internal Medicine* can be found at: www.wileyonlinelibrary.com/journal/jvim

**Disclaimer**

The Publisher, the American College of Veterinary Internal Medicine and Editors cannot be held responsible for any errors in or any consequences arising from the use of information contained in this journal. The views and opinions expressed do not necessarily reflect those of the Publisher, the American College of Veterinary Internal Medicine or Editors, neither does the publication of advertisements constitute any endorsement by the Publisher, the American College of Veterinary Internal Medicine, Editors, or Authors of the products advertised.

For submission instructions and all other information visit: www.wileyonlinelibrary.com/journal/jvim
